# Older Adult Cohousing Communities: The Lived Experience

**DOI:** 10.1093/geroni/igaa057.361

**Published:** 2020-12-16

**Authors:** Sherry Cummings, Nancy Kropf

**Affiliations:** 1 University of Tennessee Knoxville, Nashville, Tennessee, United States; 2 Georgia State University, Atlanta, United States

## Abstract

“We’re on the leading edge of the baby boomers so we don’t do anything like anybody has ever done before and that includes aging.” (Tammy, SS). This quote embodies the perspective of older adults engaged in a new housing phenomenon – older adult cohousing communities (OACCs). OACCs are designed, managed, governed and maintained by the older residents themselves. Seventeen such communities currently exist, and more are being developed by active baby boomers who are searching for more meaningful, relational and eco-friendly options for aging-in-place. The older adult cohousing movement in the U.S., which began in 2005, is small but growing quickly. However, a dearth of literature exists on this phenomenon. This qualitative study examined older adult cohousers’ lived experiences. The study employed an existential-phenomenological research method. Maximum variation purposive sampling was employed. Twelve communities were identified that represented the full range of geographic environments, structures, missions, cost and size. Interviews were conducted individually or in focus groups. In all, 73 older adult cohousers participated in the study. The Gerotranscendence Theory of Aging was used to guide the development of the structured interview questions and to organize data analysis and interpretation. Digital recordings were transcribed, and an inductive method was used to allow codes and themes to arise from the data itself. Patton’s (1990) criteria for judging themes was employed. This poster will clarify the nature, principles and structure of OACCs. Themes that emerged from the study - benefits, challenges, aging-in-place, interpersonal relationships and personal growth - will be described .

